# Conversation analysis and power: examining the descendants and antecedents of social action

**DOI:** 10.3389/fsoc.2023.1196672

**Published:** 2023-06-07

**Authors:** Mats Ekström, Melisa Stevanovic

**Affiliations:** ^1^Faculty of Social Sciences, Department of Journalism, Media, and Communication, University of Gothenburg, Gothenburg, Sweden; ^2^Faculty of Social Sciences, Tampere University, Tampere, Finland

**Keywords:** conversation analysis, power, deontics, authority, social interaction

## Abstract

Conversation Analysis (CA) tends to adopt an ambivalent attitude to the concept of power. The concept is fundamental in sociology but secondary or even disregarded in CA. A closer look at research and the conceptual foundations of CA however demonstrate significant contributions to theories of power. In this paper we aim to demonstrate and discuss these contributions, however, also arguing for an expansion of the CA approach in dialogue with sociological theories to engage in the sociological analysis of power as an essential feature of social relationships and social organization. Based on a general definition of power, as the transformative capacities of social agents in virtue of their social relationships, we discuss how power is interactionally achieved and negotiated, but also conditioned by social institutions and structures that extend beyond the contexts of situated encounters. The paper is divided into two main sections. The first section presents central contributions of CA in relation to the distinctions between power over and power to, authority as a legitimate form of power, and deontics as a key concept in the analysis of power. The second section critically considers the tendency in CA to localize power solely to actions in interaction, and to conflate structure and action, which constraints the analysis and explanations of power. We present examples of how analyses of power, grounded in CA, can be extended to account for the dynamics of social structures and realities beyond the interactional encounters.

## Introduction

Human social existence is permeated by power. It is an essential feature of social relationships and social organization in all forms of political, organizational, and institutional life. Yet, power is a concept that tends to be avoided in conversation analysis (CA). This avoidance can be clarified with reference to the ethnomethodological roots of CA. Constituting a break from the traditional social scientific approaches, ethnomethodology sought to explain social order with reference to the mundane practices by which members make sense of the world and act in it (Heritage, 1984). What is thus demanded of social science is to document “the processes by which social life is constituted rather than treating social phenomena as given objects in the world” (Hammersley, 2003; p. 755). In this paper we aim to demonstrate the significant contributions of CA to the study of power, however, also arguing for an expansion of the approach in dialogue with sociological theories.

The rhetoric of CA involves the researcher being able to “sit back and observe the structuring quality of the world as it happens” (Boden, 1994; p. 74). This idea presupposes a view in which social reality is realized in and through the publicly observable features of interaction and is in this form also researchable (e.g., Heritage, 1984; Schegloff, 2007). In this sense, CA is committed to “ontological muteness” (Gergen, 1994; p. 72) regarding those aspects of social reality that go beyond what can be observed in the participants' conduct. Similarly, CA has rejected the “bucket theory of context” (Heritage, 1987; Goodwin and Heritage, 1990; p. 286) in which pre-existing social structures are seen to determine interaction from above. Rather than seeing the context as an abstract social force imposed on the participants, CA researchers have observed how the participants actively display their orientations to the context (Hutchby and Wooffitt, 1998; p. 147). In other words, contextual features are not considered relevant for research if the participants themselves do not orient to their relevance in their publicly observable behavior (see Arminen, 2000; p. 446). In this sense, CA is permeated by a form of “agnosticism” that treats the existence of the higher-level social phenomena, such as power, as unknowable (Hutchby, 1996, 1999; p. 482; p. 86).

The focus on participants' publicly displayed orientations as the only basis for making analytic claims, combined with a general reluctance to engage in sociological theorizing, has led many sociologists to question the bearing of CA on what might be called the sociological agenda (Wetherell, 1998; Billig, 1999; Hutchby, 1999). Much of this criticism has to do with the notion of power and the ways in which power relations may affect what different people can do in their interactions with others and how they can legitimately treat their interaction partners (Burr, 2015; p. 5). For example, there are situations (e.g., sexual harassment) in which a researcher may have compelling reasons to assume that the participants' relationship is in some way fundamentally unequal or unbalanced. In these cases, a sole focus on the participants' publicly displayed orientations leaves the researcher at the risk of disregarding those ideological and cultural-historical aspects of power (Mann, 1986) that shape both their own publicly observable behaviors and their patterns of interpreting other people's behaviors (Wetherell, 1998; Billig, 1999). In this way, the capacity of CA to engage in social and societal critique is severely compromised.

Despite the traditional skepticism of CA with the notion of power, CA studies on power have recently become increasingly frequent. As we will discuss further below, this tendency seems to be particularly prevalent in applied CA—that is, in research on various institutional contexts where power relations are central, such as family therapy (e.g., Ong et al., 2021), court and police interrogations (e.g., Haworth, 2006), parliamentary debates (e.g., Antaki and Leudar, 2001), classrooms (e.g., Stephenson, 2020), and meetings (e.g., Boden, 1994). However, CA still has not really embraced sociological theorizing on power. Instead, the existing CA studies on power are published in journals primarily addressing the CA community. Furthermore, some CA researchers have emphasized that the analysis of power in sequences of talk does well without any sociological theoretical underpinnings external to CA (see e.g., Hutchby, 1996; p. 483). Instead, neighbor concepts, such as asymmetries, have been embraced more readily.

In this paper, we address the interface between CA and power, also engaging in more abstract sociological theorizing on how power can be intertwined with the local organization of action. The paper is organized in the following way. Next, we will discuss social power as a concept. We adhere to the conceptualization of power as transformative capacities of agents in their social relations (Giddens, 1984; Isaac, 1987a; Sayer, 2012), which we find particular relevant, and essentially consistent with, the action in context approach in CA. Thereafter we discuss the capacities of an individual to act in virtue of social relations in sequences of social interaction, specifically drawing on the distinction between *power over* and *power to*. Thereafter, the remainder of the paper is divided into two main sections. In the first, we will consider *authority* as a form of power, enacted in virtue of social and institutional relationships. In the second section, we rely on insights offered by critical realism with regards the agency/structure relation and the stratification of social reality and discuss some implications to the contribution of CA to the analysis of power. Finally, we suggest a few hypotheses to be tested in future CA-informed sociological studies on power.

## The concept of power

As a concept, power has been widely theorized and discussed in social science (Clegg, 1989). Power is analyzed as underlying features of social relations and structures in general and shown to exist in different forms; in actors' capacities to influence and control; in dominance and dependencies; in authority, coercion, and access to means of violence. As Sayer (2012; p. 81) has argued “there is no such thing as power-as-such.” Power is always the power of actors in social relations. Moreover, power is not a particular resource but performed through various resources mobilized by actors to achieve the goal of the action.

Weber (1978; p. 53) presented what has become a seminal definition of power. Power refers to “the probability that one actor within a social relationship will be in a position to carry out his own will despite resistance ….” Weber's perspective on power includes two basic components, elaborated in theories on power. First, power is about agency *within* social relations. Second, power refers to what actors *can do* but also *the influence over others* which may manifest in domination as well as resistance. Following the literature, we will refer to this as the *power-to* and *power-over*.

Power has been described as a “transformative capacity” (Sayer, 2012; p. 181), a capacity of agents (individuals, groups, and organizations) to influence and make a difference in the world, on social and material conditions and concrete course of events (Giddens, 1984; p. 14; Isaac, 1987a; p. 21). In sociology, the primary object of analysis is *social power*, focusing on capacities in the social domains of reality. This is well articulated in the following definition to which we adhere: Social power refers to “the capacities to act possessed by social agents in virtue of the enduring relations in which they participate” (Isaac, 1987a; p. 22). This locations of power in agency, is essentially different from, for example, Foucault's theory of power, suggesting that power is best understood as being everywhere, operating independent of agency, diffused in discourses constituting agents (Foucault, 1991, 1998).

Isaac's definition of power distinguishes several critical aspects. In defining power as *capacities* of *social agents* we avoid the limiting behaviorist account, where power tends to be reduced to actual behaviors and their results on the behaviors of others (Isaac, 1987b; p. 76). The analysis instead focuses on capacities to make an impact and to achieve intentions or goals, conditioned by the social relations and normatively constituted activities in which actors participate. Capacities are largely enduring (Sayer, 2012; p. 186), mobilized, differently exercised, and negotiated in concrete actions in interaction. Capacities thus exist even when not activated. Though, as Isaac (1987b; p. 81) notes “a social power that is never exercised can hardly be said to exist”. The idea of latent capacities for exercise of power and resistance, possibly shaping what happens in interactions, seems contradictory to the CA idea of reality as realized in displayed and observable behaviors. We will however argue that the idea of enduring and latent capacities is a prerequisite for understanding the performance of power in social interaction.

It is agents, and not structures, who have power. However, agents have power only *in virtue of* social relationships. This applies to governments and authorities dependent on public support and legitimacy to rule and get things done, as well as individuals exercising power within established roles and relations in everyday life. The *virtue of* is a main object in the analysis of social power, to explain the dynamics of power, inequalities in power, and the enactment (and the lack) of capacities. Actors rely on and invoke authority, a legitimate power to influence others, in virtue of roles and identities in institutionalized activities, but also depending on whether others ascribe legitimacy to their actions. Actors' capacities and resources to influence their own situation and make a difference in society are constrained and enabled by their positions in social structures. Resources are, as Giddens (1984; p. 15) has noted, “properties of social structures,” invoked and drawn upon by agents in social interaction. Analyses of power thus elucidate the capacity/relationship nexus in normatively constituted activities and layers of social reality; in situated interaction, institutional arrangements, and societal structures.

As pointed out above, social theory identifies two basic forms of power: *power-over* and *power-to*. The distinction further clarifies the conceptualization of power presented above. *Power-over* refers to relations of dominance and control; actors' abilities to govern the situation and action of others, to make others act in a way they would not otherwise have done (Pitkin, 1972; Isaac, 1987b; Morriss, 2002). A social relation in terms of *power over* is thought of as necessarily conflictual and is mostly used as a synonym for domination (VeneKlasen and Miller, 2002). *Power-to*, in contrast, refers to the capacities to accomplish actions and make a difference, by virtue of the social relations (Morriss, 2002). As it is not defined with reference to the consequences of the individual's actions for others, it is regarded as a consensual and intrinsically legitimate instantiation of power. Some accounts of power suggest that both *power-over* and *power-to* should be included in any comprehensive understanding of power. For example, Pansardi (2012) argued that both *power-to* and *power-over* should be seen as relational concepts—that is, as two aspects of a single, more general concept of social power. As put by Pansardi (2012), “power to and power over refer to the same social facts, they both consist in the changing of someone else's incentive structure and in the obtainment of a specific outcome, no matter whether they refer to something I can do by myself, having obtained the non-interference of others, or in the specific product of someone else's action” (p. 84). However, *power-to* does not imply a *power-over* in the form of domination (Sayer, 2012; p. 183). The power to accomplish certain actions in social relations have different outcomes depending on the actions of others. Domination may be avoided and resisted.

## Capacities to act in virtue of social relations in sequences of social interaction

From the perspective of CA, the notions of *power-over* and *power-to* are essentially about the participants' capacities to act in virtue of social relations in sequences of social interaction. *Power-over* can be identified based on the constraints that a participant imposes on another participant's freedom of choice, which allows them to achieve interactional goals and aims. Some of these constraints have to do with actions in general, which also encompasses those that go beyond the interactional encounter, while other constraints deal specifically with what happens in the interaction here and now. In contrast, *power-to* is about the capacities of the individuals to act on their own. In social interaction, *power-to* may be seen in the extent to which a participant is able to implement social actions in a sequence and to act within the currently existing sequential constraints.

From the perspective of constraints imposed on action in general, *power-over* may be associated with the class of directive speech acts including orders, commands, and requests. Imperatives, for example, represent the most stereotypical way of giving orders and commands to another person (Craven and Potter, 2010; p. 442) and thus constitute a central practice for exercising *power-over*. It is worth stressing, however, that imperatives can also be used to perform actions that have little to do with power over, such as instructing someone toward the means of achieving something that they themselves want to pursue or making an offer or an invitation (Sorjonen et al., 2017). Another stereotypical way of exercising *power-over* other people's actions involves the use of deontic modality, as the modal verbs such as *ought, must*, and *should* can be used by a speaker to impose constraints on another person (see Sterponi, 2003; Curl and Drew, 2008). However, again, the mere existence of a deontic modal verb in an utterance is not enough to make the utterance count as an instance of *power-over*. For example, pieces of advice from a friend are often likely to contain such verbs (*you must see a doctor*), but it is the person themselves who may still be entitled to choose freely whether to follow that advice or not.

From the perspective of people's possibilities to act in the interaction here and now, *power-over* may be seen to encompass any dominant interactional behavior that is unresponsive to other people's concerns and constraints their possibilities to address them. For example, someone may talk much more than their co-participants, determining the topics of conversation and imposing their views on the things talked about (Linell et al., 1988; Hutchby, 1996, 1999), while others cannot but try to cope with the type of power that is exercised over them. Some forms of *power-over* may also be identified in situations in which participants position themselves as more competent and knowledgeable than others (Thornborrow, 2002), in this way seeking to influence their co-participants' beliefs, attitudes, or actions. Persuasion as *power-over* may operate, for example, through explanatory accounts (Heritage, 1988; Houtkoop, 1990), strategical displays of emotion (Fitch and Foley, 2007; Nikander, 2007), and through other discursive, rhetorical, and argumentative practices by which people manage to silence others.

*Power-over* becomes also visible in those situations in which control over the agenda of the interaction is in the hands of a specific person. This is typical in various institutional interactions, in which the participants construct an asymmetrical turn-taking systems that endow them with quite inequal amounts of freedom in terms of their talk (Macbeth, 1991; Kendall, 1993). Control over agenda is unequally distributed also in various group interactions, such as meetings (Drew and Heritage, 1992; Boden, 1994; Angouri and Marra, 2011). Although the role of the chairperson in a meeting may be considered merely as an effective practice to manage turn-taking in a complex multi-person setting and to facilitate joint decision-making on topics of great significance, research has shown that the role of the “mere” agenda manager easily slides into one that also encompasses control over the content of the decisions to be made (see e.g., Valkeapää et al., 2019; Stevanovic et al., 2022). Likewise, many social institutions would not operate smoothly and fill their purposes in society without power relations (see e.g., Pilnick, 2022), but this does not mean that we should ignore the existence of constraints on people's freedoms caused by the exercise of *power-over* in these settings.

The notion of control over the local agenda of interaction is deeply intertwined with a phenomenon of still more “local” nature—that is, that of the “conditional relevance” of a specific responsive action upon the occurrence of a specific initiating action (Schegloff, 2007; pp. 20–21). This principle is held together by accountability: should an adequate responsive action be missing, an account for the omission or failure will be required (Heritage, 1984; pp. 245–253). Notable, the notion of conditional relevance refers to utterances or actions and their relationship with one another—that is, it is about “items” and not people (Schegloff, 2010; p. 39). However, starting from Stivers and Rossano's seminal attempt to tease apart the components of conditional relevance (Stivers and Rossano, 2010a,b), and continued by Heritage's notion of the “epistemic engine” as the driving force of sequences (Heritage, 2012; see also Drew, 2012), an opportunity space has been opened to shift the focus of interest in conditional relevance from items—that is, actions and their relationship with one another—to the *actors* who produce these items. Indeed, the items do not operate by themselves. Instead, it is the *participants* producing the initiating actions that put their co-participants under the normative constraints either to produce relevant responsive actions or to become accountable for not doing so (Stevanovic, 2018). In other words, conditional relevance operates based on *power-over*.

From this perspective of CA, *power-to* realizes in different forms depending on the sequential position. In the context of sequence-initiating actions, *power to* realizes as the capacity to carry out powerful actions, such as announcements of unilateral decisions, without an orientation to a need to get others' approval for them (Stevanovic and Peräkylä, 2012). *Power-to* also involves the capacity of a person to demand specific types of responses from others and the possession of effective ways to deal with others' possible reluctance in providing these responses. In the context of responsive action, *power-to* can be observed in people's ways of creatively dealing with the constraints imposed on them by other. Possibilities to resist constraints without becoming accountable for doing so vary depending on the person's position, involving, for example, strategical ignorance or claims of ownership of the action imposed (Stevanovic, 2021a). In a sequential third position, *power-to* becomes visible in a person's capacity to stick to one's initial line of action, which may realize, in its most simple form, as sequential deletion or, in its most complex form, as an integration of the other people's resistance to the initial line of action.

In sum, in CA, *power-to* and *power-over* are studied as social interactional phenomena. The effects of actions in social interaction depends on the understanding and responses of others. Hence, *power-to* is not an ultimate capacity but (at least potentially) open to resistance, even within social structures of supremacy and subordination and institutionalized relations of inequal capacities and resources. Giddens (1984; p. 16) referred to this as the “dialectic of control.” *Power-over* in social interaction relies on forms of dependency, which also offers resources for the subordinated to respond and influence the activities and relations to the superior. Thereby, *power-to* and *power-over* are deeply intertwined. On one hand, the opportunity context which constitutes *power-to* is made up of specific social relations of *power-over*. In other words, if you determine the actions of other people, you can also achieve a lot through these people, which further increases your own individual capacities. On the other hand, if you determine your actions unilaterally, others will need to adjust their own actions accordingly. Therefore, there is not always a need to distinguish between these two aspects of power. However, as we will demonstrate further below, this distinction can help shed light on certain social interaction patterns that would otherwise be hard to make sense of.

## Authority in virtue of social and institutional relationships

Authority is a basic form of power, where governing and directives are followed and treated as *legitimate*. In short, authority is legitimate power. The understanding and approval of the exercise of power as legitimate is thus a quality of social relationships that give actors the capacity to determine the actions of others. In sociological theory, authority is typically contrasted to power exercised by coercion and violence.

In social theory and political philosophy, authority has been described with reference to multiple distinctions. Most famously, Weber (1978) distinguished three sub-types of authority: “legal” (the approval of legislations and the right of actors to issue laws and directives), “traditional” (acceptance based on habits and traditions) and “charismatic” (a willingness to follow based on the trust in the leader's extraordinary personal qualities). Other distinctions have described the concept as involving two sides to it. One widespread view has distinguished between authority “by fact” (*de facto*) and authority “by law” (*de jure*) (Peters, 1967). Other conceptualizations distinguish between “authority over belief” vs. “authority over conduct” (Lukes, 1978) and “epistemic authority” vs. “deontic authority” (Bochenski, 1974). Some of these distinctions deal with the specific *sources* of power in virtue of which a person is an authority (e.g., law, as it the case in “legal authority”), while others are more about the ways in which authority *realizes* in practice (e.g., as control over decisions, as is the case in “deontic authority”).

CA has contributed to the analysis of authority both in terms of (1) its sources and (2) its ways of realization. In addition, and quite prevalently, CA has demonstrated the ways in which (3) authority is negotiated—claimed, justified, approved, or resisted—in turn-by-turn interactions. Although these different aspects of authority are necessarily intertwined in any empirical analysis of authority in a specific context, we maintain—and hope to be able to demonstrate below—that it is still theoretically beneficial to keep these aspects conceptually separate.

### Sources of authority

As for the sources of authority, it is grounded in realities structuring distinctive relationships in institutional contexts. Parsons (1939; p. 461) distinguished between *authority in virtue of a specific competence* and *authority in virtue of office*. The two forms of authority are both, but in different ways, “functionally specific” and legitimized by institutional systems such as the education and credentialing of doctors and the laws and rules that give officials rights to make decisions in specific areas.

*Professional authority* is a type of authority extensively analyzed in sociology, including contributions from CA (Heritage and Clayman, 2010; Stivers and Timmermans, 2020). The concept was introduced by Parsons (1939, 1951) in his theory explaining the central role of professions in the increasingly complex and differentiated modern society. Much of professional authority can be clarified with reference to *authority in virtue of a specific competence*. Professions are assumed to represent rational values such as neutralism and universalism, and a specialized technical competence required to carry out the work. Professional authority is thus based on, and always restricted to, a particular field of achieved knowledge and the epistemic regime of academic expertise. As Parsons (1939; p. 460) notes, this type of authority has “a peculiar sociological structure,” in not being grounded in a general relationship of superiority, but “the technical competence of the professional man,” who in this relationship also have power over people who would be otherwise superior in status and position in society. One important subtype of professional authority, which Parsons discussed extensively, is *medical authority*.

However, professional authority is also largely about *authority in virtue of office*, which refers to the power to do things and to command others in acting on behalf of an administrative office. This is the case, for example, in public agencies and welfare services, in which officials regularly meet clients in conversations about applications, eligibility and decisions about various services, social support and economic benefits (Bruhn and Ekström, 2017). In these institutional contexts, officials act on behalf of laws, regulations, and routines. This is evident when officials justify decisions with references to regulations, as in these examples from a study on the Swedish Board for Study Support: “No we have no ability to do that,” “We cannot do that, we have our rules to follow,” “We have hard restrictions about that” (Bruhn and Ekström, 2017). The justifications are produced in a context where the official declines a request about a reduced repayment of a debt. Note that officials talk on behalf of a “we” (the office) and present the decline as non-negotiable. These officials act, and respond to clients' requests, within a system of laws and detailed rules and routines.

The sources of authority can, however, be more multiple than implied by the Parsonian distinction. For example, this is the case for what Clayman has defined as the *question authority*. The legitimate right to ask questions is fundamental to the performance of institutions in modern society including judiciary, police, social work, health care, social research, and journalism (Antaki et al., 2002; Ekström et al., 2006; Haworth, 2006; Clayman et al., 2010; Iversen, 2012; Danermark et al., 2019). The question authority is central in journalism, in which the intended interviewees “should make themselves accessible” to interrogations, accept and try to answer the questions that the interviewer deems relevant to ask (Clayman, 2002; p. 198). Importantly, however, CA has provided evidence that journalists claim authority neither in virtue of a specialized competence, nor in virtue of office. Instead, the legitimacy of questioning is grounded on *authority in virtue of institutionalized practices*, which in this case have to do with the practices of interviewing in the media, the assumed norms and values of professional journalism in liberal democracy, and a related “unspoken contract” between journalists and public figures (Clayman and Heritage, 2002a; Ekström et al., 2006; p. 29). As Carlson et al. (2022; p. 120) note, in a study of Trump's and the right-wing populists' attacks on news media, and the undermining of journalists' authority in for example press conferences, this is particularly challenging for journalism as they have “few means of enforcing their authority outside the appealing to norms to support their work.”

### The realization of authority

As for the ways in which authority realizes in everyday life, it becomes central do bear in mind those definitions of authority that distinguish between the authority in the field of *knowledge* and the authority in the field of *action* (Bochenski, 1974; Lukes, 2005). In CA, these two areas in the application of authority are commonly referred to as *epistemic authority* (e.g., Heritage and Raymond, 2005) and *deontic authority* (e.g., Stevanovic and Peräkylä, 2012). Research in CA has shown how participants orient to their own and each other's epistemic rights (access to knowledge) and deontic rights (rights to determine action) in the ways in which they design their utterances and respond to those of their co-participants.

The distinction between epistemic authority and deontic authority can be clarified, for example, with reference to *medical authority*. Parsons who used the physicians as an example of how professionals have the right to ask questions, make judgments, and prescribe actions that the layman/the patient is expected to accept and follow ‘on authority' (Parsons, 1939; Heritage and Clayman, 2010). To use the terminology of Searle (1976), epistemic authority is about getting the “words to match the world”, deontics is about getting the “world to match the words” (Stevanovic and Peräkylä, 2012). Thus, for example, doctors' right to make diagnoses are clearly a matter of epistemic authority, while treatment recommendations and medicine prescriptions are a matter of deontic authority (e.g., Landmark et al., 2015; Lindström and Weatherall, 2015). Just like in the case of medical authority, concerns of knowledge are often only the first step toward those concerns that have to do with actions based on what can be known about the current matter at hand. Therefore, concerns of power are most straightforwardly linked to the notion of deontic authority—though, importantly, power has often been noted to operate in the disguise of knowledge and epistemic authority (Stevanovic, 2013, 2015, 2017; Landmark et al., 2015; Lindström and Weatherall, 2015; Svennevig and Djordjilovic, 2015).

Although rights to determine action are an omnirelevant aspect of social interaction, deontic authority has been specifically investigated in the contexts of specific activities and interactional phenomena. These include directive instruction (Henderson, 2020; Frick and Palola, 2022), support work (Antaki and Webb, 2019), joint decision making (Stevanovic, 2012), participatory democracy (Magnusson, 2020; Wåhlin-Jacobsen and Abildgaard, 2020), leadership (Clifton et al., 2018; Van De Mieroop, 2020), agenda management (Stephenson, 2020), and teaching development (Ripatti-Torniainen and Stevanovic, 2023). In all these contexts, the rights to determine action may concern future action (*distal deontics*) or joint action unfolding locally in the encounter (*proximal deontics*), these two temporal fields being often intertwined in complex ways (see e.g., Stevanovic, 2015; Clifton et al., 2018; Magnusson, 2020; Stephenson, 2020; Van De Mieroop, 2020; Stevanovic et al., 2022).

### Negotiations of authority

As pointed out above, CA has been specifically influential in showing how authority is negotiated—claimed, justified, approved, or resisted—in turn-by-turn interactions. Most generally, CA research on deontic authority has focused on how participants' deontic rights are oriented to and drawn upon, as observable in the ways in which the participants design their actions and organize them as sequences of action. A typical example involves a first speaker making a stronger claim of deontic authority than the recipient is willing to validate, which leads to the recipient manipulating the terms of their responsive action. In their responses, the recipients may claim for themselves a greater share of power and “ownership” of the participants' line of action than what was initially offered to them (see e.g., Stevanovic and Peräkylä, 2012; Stevanovic and Monzoni, 2016; Keevallik, 2017). The recipients may also selectively orient to or disregard either the distal or proximal aspects of deontic authority, which enables most intricate negotiations of leadership, expertise, and power (see e.g., Van De Mieroop, 2020; Stevanovic, 2021a).

Another example of a standard way in which people commonly negotiate their authority relationship is about whether some constraint has been imposed by external forces or by the co-participant. People tend to avoid personal imposition and thus often refer to external forces. Thus, for example, in the context of aggressive journalism, a particular practice to justify adversarial questioning is, for example, to invoke knowledge about people's interests, in which case the journalist merely enacts the role of a watchdog of society, who works on behalf of the public (Clayman, 2002). Analogous phenomena appear also in the field of medical authority, where doctors commonly invoke external facts as persuasion strategies to deal with patients' resistance (Stivers and Timmermans, 2020) and present public evidence for their diagnostic statements (Peräkylä, 1998), as well as in the field of education and teaching, where instructions are often constructed ambiguously as for their epistemic vs deontic nature (Stevanovic, 2017). What is common to these strategies is the mitigation of deontic authority, without yet compromising epistemic authority.

Most importantly, CA has shown that the legitimacy is never unconditional, but it must be achieved (Clayman, 2002; p. 198). In focusing on the design and sequences of turns of talk in medical encounters, CA has shown how authority not only presents itself in diagnoses and treatment recommendations delivered as authoritative, but also in how that authority is further constituted and approved in patient's responses, as well as in how certain responses intrude to this authority (Stivers, 2005; Heritage and Clayman, 2010; p. 159). Similarly, while politicians may approve the question authority of journalism by demonstrating a willingness to answer even critical questions and questions that cannot be answered (Ekström, 2009a), the legitimacy of journalism may also be challenged within (and outside) interviews. Politicians may undermine the journalist's status by criticizing the questions asked, holding the journalist accountable for the assumptions made, or simply ignoring (even disdainfully) certain journalists in public press conferences (Clayman, 2002; Ekström, 2009b). Likewise, even in the highly standardized institutional context such as public agencies and welfare services, in which power takes the form of *authority in virtue of office*, authority does not assume predetermined forms. When an official says: “well I could make an exception here”, we may observe authority that is flexible in how to apply the regulations in particular situations (Bruhn and Ekström, 2017; p. 208).

### Toward the separation of a priori and a posteriori

In sum, CA has contributed to a better understanding of the sources of authority, its ways of realization, and the ways in which it is negotiated in turn-by-turn interactions. As these aspects of authority are necessarily intertwined in any empirical analysis of authority in a specific context, CA studies on authority have seldom tried to keep these aspects conceptually separate. This means that what is considered at a given moment of interaction a precondition of interaction (*a priori*) and what is considered as its outcome (*a posteriori*). This limits the opportunities to analyze power in interaction.

Maintaining that it is theoretically beneficial to keep the *a priori* and *a posteriori* aspects of power conceptually separate, we will now draw specific attention to the distinction between *deontic status* and *deontic stance*, which has been recently used in the CA research on deontic authority. *Deontic status* refers to the *latent capacity* of a person to do so, independent of whether the person has publicly claimed it or not, while *deontic stance* refers to the publicly displayed rights to determine action (cf. the distinction between capacities and activation in section two above). The distinction is important in that the deontic stance that a participant takes in and through the publicly observable features of action-design may be *congruent* or *incongruent* with the participant's deontic status (Heritage, 2013; Stevanovic, 2018). Expectedly, people generally want to design their publicly displayed deontic stance to be congruent with their deontic status (Heritage, 2013a; p. 570), as a strongly authoritative deontic stance without the deontic status backing it up runs at the risk of being challenged by others (Wåhlin-Jacobsen and Abildgaard, 2020; p. 47). However, various types of deontic incongruencies are also common.

One example of a deontic incongruence is the so-called “first position downgrading incongruence” (Stevanovic, 2018). It involves the first speaker publicly displaying a low deontic stance while relying on their high deontic status to achieve the desired interactional consequences. The capacity to design one's utterances with this type of deontic incongruence is *power* in its most evident form—there is no need to command or order. The mere position of power allows the person to exercise both power-over and power-to, simply by virtue of others seeking to comply with the person's wishes even when these have not been expressed. However, this type of deontic incongruence is also a risky endeavor. For example, a mother may first seek to direct her child with a soft reminder that is oriented to the child's own desires and autonomy (*d'you wanna go pop your toothbrush back and give it a try*). Yet, if the child does not comply, the mother may ultimately need to reveal the real nature of her action: in reality, the child has no choice but to comply (see Henderson, 2020).

From this perspective, the analysis of power is essentially about considering the ways in which participants give weight to each other's deontic statuses and deontic stances. Instead of always needing to claim their deontic rights (*deontic stance*) a powerful participant may also trust in their co-participants being aware of and considering these rights anyway (*deontic status*). As pointed out by Tomasello (2008), in all human social interaction, the relationship between the participants' overt interactional conduct and the intersubjective context of the interaction is *complementary*: “as more can be assumed to be shared between communicator and recipient, less needs to be overtly expressed” (p. 79). In this sense, deontic status as an interactional resource is not equally available for everyone. This notion, in turn, opens a way to link the descriptive considerations of power to the normative notions of inequalities and to use them as a tool of social and societal critique.

## Beyond the local negotiation of power in talk and interaction

In CA, power is localized primarily to actions in interaction. CA does not ontologically exclude macro- and institutional features of society but claims to contribute by linking interaction to such higher-level features in the analysis of power (Hutchby, 1999; Wooffitt, 2011). However, the linking is assumed to happen only within interaction, or as Hutchby (1999; p. 86) argues “high level features of society are only instantiated in and through talk.” We believe this constrains the analysis of power, and the relevance of CA, regardless of whether it is perceived as an ontological or methodological position. The social structures in which power is exercised are not solely interactional phenomenon. The concept of social structure is thoroughly discussed in ethnomethodology and CA (Boden and Zimmerman, 1991). In these theoretical traditions, social structures are conceptualized as something people do, as practical accomplishments. Social structures are shaped in and through patterns of talk and interaction. In institutional settings, structures are, for example, shaped in actors' orientations to institutional identities and participant roles. The empirical evidence of these social mechanisms is extensive. However, the approach also tends to conflate structure and action, and reduce their different properties and dynamics (Danermark et al., 2019).

The structures by virtue of which actors have power to determine action—both in the sense of *power-over* and *power-to*—partly exist *outside* agents' actions in interaction. They are not invented in moments of interaction. And as argued above, actors have capacities and deontic rights also when not activated in moments of interactions. This idea of structures as already existing for actors seems to be assumed in CA, when referring to the *pre-allocation* of roles and resources in institutional interaction. Moreover, what makes social structures enduring is not only the normative orientations to patterns in interaction, but also their manifestation in institutional arrangements outside interactions, in formal organizations and processes, legal and regulatory documents, allocation of resources and so on. As Heritage (1997, p. 223) notes, CA is concerned with how such institutional realities are “evoked, manipulated and even transformed in interaction”, but don't assume that institutional realities are “confined to talk”.

Following the philosophy of Critical realism (Archer, 2000, 2017; Danermark et al., 2019) presents a model explaining structures and agency as mutually dependent yet qualitatively different phenomenon. It is actors, and not structures, that have agency. Actions take place within enabling and constraining social structures, which exist as an outcome of human actions. However, by taking emergence into account, and introducing a temporal dimension in the analytical model, Archer clarifies that actions in virtue of structures and structures in virtue of actions are not moments of the same process (Danermark et al., 2019; p. 81). Archer proposes an analytical model with three phases in a cycle. The *first* phase consists of the enduring social structures emerging from previous generations of agents and social interactions. The *second* phase consists of moments of actions and social interaction. This is the phase of agency. Only people have capacities to make a difference in social life, however conditioned by the preceding, already existing, social structures. Hence, structures are not created in the moments of interaction, but evoked, realized, negotiated, or manipulated. The *third* phase consists of the reproduction and transformations of social structures (e.g., positions and roles in social relations and institutions) with implications for the structures preceding future actions (phase 1 in the cycle). This analytical model is of course an abstraction. Archer (2000, 2017) substantiates the model in extensive analyses. In this context, we introduce the model to frame and expand the analysis of power in CA. In what follows, we discuss two dimensions: the *temporal*- and the *multi-layered* analyses of social power.

### The temporal dimension: preceding structures, deployment, and transformations

CA is at its best in the analysis of what happens in the interactional sequences produced in clearly delineated interactional encounters, the boundaries of which coincide with the start and end of the videorecording. Extending the analysis of interaction to reach the social world beyond the encounter is therefore a challenge that needs to be considered from two different angles: (1) from what precedes the interactional encounter (*antecedents*) and (2) from what follows the encounter (*descendents*). In both cases, it is worthwhile to explore the critical realist insights regarding the separation of structures and agency (Archer, 2000, 2017; Danermark et al., pp. 76–79). In the context of social interaction, agency is basically about any publicly recognizable action implemented by the participants in an interactional encounter, while structures are those features of the social world that precede and follow the implementation of each such action. This means that any sequence of interaction is a locus of constant deployment of structures, and this is also something that can be investigated empirically through traditional CA methods.

The consideration of the antecedents of action is connected to the discussion on action formation (Levinson, 2013). CA researchers have commonly dealt with the phenomenon by focusing on various “social action formats” (Fox, 2007)—that is, regularly patterned clusters of publicly observable resources that are deployed to convey specific actions, such as offers (e.g., Kärkkäinen and Keisanen, 2012), proposals (e.g., Stevanovic, 2013), and complaints (e.g., Ogden, 2010). Furthermore, the complex ways in which the verbal dimension of the participants' conduct is embedded in the material and embodied elements of the situated courses of action have been referred to as “multimodal gestalts” (Mondada, 2014), “social action formats” (Rauniomaa and Keisanen, 2012), or “multimodal action packages” (Lilja and Piirainen-Marsh, 2019; Stevanovic, 2021b). Instead, less focus has been paid to the considerations of the broader structural features, such as power relations, that inform the design of and accountabilities associated with specific actions (Stevanovic and Peräkylä, 2014; on requests, however, see Antaki and Kent, 2012). While systematic empirical claims about the precise role of these contextual features on action formation are challenging to make, there is no reason to believe that action formation would operate independently of these features.

Extensive research on interaction in institutional contexts has shown a distribution of roles and resources that is “characteristically asymmetrical” (Drew and Heritage, 1992; p. 47), “pre-inscribed” (Thornborrow, 2002; p. 4), and thus precedes the moments of talk and interaction. The participant roles, enacted within certain turn types, are associated with rights and capacities to influence the activity. The preceding structures are indicated when the roles are taken for granted at the beginning of the interaction, without being described or justified, and when deviations are noticed and handled by the participants as such. As summarized by Thornborrow (2002; p. 4) “institutional discourse can be described as talk which sets up positions for people to talk from and restricts some speakers' access to certain kinds of discursive actions.”

The antecedents of action may also be considered from the perspective of wider cultural and historical developments. In his study on doctor's diagnostic statements, Peräkylä (1998) saw the doctors to coordinate the location and design of their diagnostic turns to preserve the accountability of some aspects of the grounds for their diagnoses. While a plain assertion of diagnosis would convey a high degree of institutional power and authority, the doctors used such turn design only when the diagnostic statement was produced immediately after the examination. When this was not the case, the doctors incorporated references to the evidential basis of the diagnosis or explicated that basis. In other words, the diagnostic statements were not presented from the position of an unconditional authority based on the doctor's superior institutional status. Peräkylä discussed his findings with reference to profound changes in doctor-patient relations during the last decades of the twentieth century, suggesting that the doctors' consistent orientation to their accountability for the evidential grounds of the diagnosis is a historically new phenomenon.

Historical development has also been considered in the context of broadcast news interviews (Clayman and Heritage, 2002a) and presidential press conferences (Clayman and Heritage, 2002b; Clayman et al., 2006, 2007, 2010). These studies identified a rise of an increasingly aggressive journalism from the 1970's to the end of the twentieth century. As potential reasons for the trend the authors, for example, referred to the heightened skepticism toward the president in the face of the Vietnam war and Watergate scandal, as well as to a more general propensity to monitor presidential performance with respect to the economy. This overall lack of trust led to the journalists becoming less inclined to accept presidential pronouncements and policies at face value and more prone to challenge presidents and hold them accountable for their actions (Clayman et al., 2010).

Long-term changes are also linked to “shift in the normative culture of journalism” (Clayman and Heritage, 2021; p. 232). Changes have been observed in the journalists' detailed practices in designing questions, which have gradually become to indicate more initiative, directness, assertiveness, and adversarialness, and less deference to the president (Clayman et al., 2006). The emergent forms of questioning have been described as “materialized” resources and practices “added to or subtracted from the journalist's repertoire” (Clayman and Heritage, 2021; p. 233). Hence, the longitudinal research on journalism indicates that structures in the form of pre-existing resources, related roles, and question authority, are reproduced but also transformed as an outcome of interactions. More recently, journalists' authority in using such resources in press conferences has been seriously challenged by Donald Trump in particular, and similar by far-right populists in other countries (Wodak et al., 2021). Politicians challenging the legitimate right of journalists to ask certain questions is nothing new (Clayman, 2002; Ekström, 2009a; Clayman and Heritage, 2021). However, the established norms in the interaction seem to be more dramatically violated in the era of far-right populism.

In the most general terms, long-term changes in the antecedents of social action may also be observed in all the ideological and cultural-historical aspects of power (Mann, 1986) that shape participants' publicly observable behaviors (Wetherell, 1998; Billig, 1999). Here, power influences the preconditions of social action by shaping access to information and cultural objects and possibilities to express views on them, opportunities to engage in various types of social actions in various types of contexts, and norms and ideals regarding the practices of bodily expression, language use, and social interaction in general (e.g., Engelstad, 2009; Nguyen and Nguyen, 2022).

Like the *antecedents* of social action, also its *descendents* must be considered on multiple timescales. As described in our prior discussion on *power-over* and *power-to*, accountabilities are tied to social power relations—structures that serve as fundamental antecedents of social action in interaction. Simultaneously these structures are descendents of social interaction. On the shortest timescale, they show in the relations between the different ways of constructing social action and the subsequent sequential development of interactions. For example, as demonstrated by Robinson (2006), doctors' social enquiries at the beginning of medical consultations may vary in terms of their turn design (*How are you doing?* vs *How are you feeling?*), which are associated with different types of patient responses (*Fine* vs *Much better, I feel good*). In addition to the immediate sequential consequences, many social actions are directly bound to longer-term consequences. This is the case, for example, in decision-making interactions, in which every decision involves a “commitment for future action” (Huisman, 2001; p. 70) and the capacity to avoid such commitment, in turn, may be regarded as a specific type of display of power (Stevanovic, 2021a).

The descendents of social action also include transformations of social relations. For example, as people attribute much value to the sharedness of information, their ways of referring to persons, places, objects, and events are not only about the efficiency of communicational, but about constructing the degree of distance or intimacy in a relationship (Enfield, 2006). Furthermore, members of certain communities may treat their “ownership” of certain forms of knowledge as the defining characteristic of their community (Sharrock, 1974; Knorr-Cetina, 1999), in which case successes or failures in specific types of knowledge displays may have drastic social consequences. Similarly significant social consequences can also be assumed to exist with reference to successes or failures in the displays of power and deontic authority.

To investigate the development of the deontic facet of social relations over time, some researchers have adopted a longitudinal CA perspective (Pekarek Doehler et al., 2018; Deppermann and Pekarek Doehler, 2021). Investigating the trajectory of a relationship between colleagues in a scientific laboratory, Jones (2023) showed that, at the beginning of their relationship both participants seem to orient to their own and each other's unilateral rights to determine their own actions. Over time, however, the participants started to intervene in each other's actions in an increasingly straightforward manner, also giving their co-participants access and possibilities to do so. Thus, instead of gaining in unilateral “power to,” the area of this capacity became *narrower* over time. However, the area of those actions in which the participants were accountable to each other, became *broader*: both participants became to have increasingly more “power over” in relation to each other. Such transformation of power relations was observable in the minor details of the participants' conduct, as they carried out their routine activities.

Again, in the most general terms, long-term changes in the descendants of social action may be observed in all the ideological and cultural-historical aspects of power that shape participants' patterns of interpreting other people's behaviors—for example, as appropriate or inappropriate. Intriguingly, language itself is deeply intertwined with the accountabilities associated with its usage. This paradoxical phenomenon can be seen, for example, in the observation that—until recently—many everyday manifestations of sexism have usually gone unnoticed as “natural” conduct, while novel terms like “mansplaining” have begun to gain ground in shaping normative expectations of appropriate conduct and thus to change the configurations of power within society (Joyce et al., 2021).

### Multi-layered approach to social power

The explanation of social power develops when the analysis of actions in interaction is related to social structures that go beyond the interaction. Social agents have capacities to influence and control, to achieve intentions or goals, in virtue of social relations constituted not only in situated interactions. The unevenly distributed access to resources for people in different social positions and roles—such as employer and employee, official and client, property owner and tenant, police and criminal suspect—is grounded in enduring, cultural and material, structures and institutions (Danermark et al., 2019; p. 84). However, as research in CA has made clear, power realizes not only in these wider social/institutional roles or identities, but also in the discursive/participant roles that are tied to the interaction (e.g., interviewer and interviewee). Thornborrow (2002; p. 35) argued that “any detailed analysis of power in interaction … needs to be informed by an account of context, the social relationships it sets up between participants, and speakers' rights and obligations in relation to their discursive and institutional roles and identities.” What we are now pointing toward is, of course, a complex of research agendas and theoretical discussions. In what follows, we will limit ourselves to illustrating the multi-layered analysis of social power with a few examples related to CA research on institutional interaction.

Actors' power in their institutional roles is determined by institutional arrangements and activities outside the interactions. Exogenous conditions affect the power that can be exercised in the interaction. CA research, for example, has shown how interviews with clients are used in welfare administration to obtain information as a basis for decisions on eligibility for services and economic support (Ekström et al., 2019). Interview practices have been proven to be crucial to the information the client provides about their situation. Practices beyond the interactions, grounded in institutional routines and regulations, however, has also shown how information from conversations with clients is devalued in relation to, for example, medical reports when the assessments and decisions on eligibility are made without the clients' involvement.

Corresponding combinations of practices within and outside interactions, regulated by institutional routines and laws, also apply to other contexts. CA research on police interviews has shown how power is negotiated and shifts as the interviews unfold. Capacities to act are grounded in pre-inscribed institutional asymmetries in participant roles and resources. Although the interviewee's power is restricted, they mobilize resources and challenge the interviewer's power to direct the conversation (Thornborrow, 2002; Haworth, 2006). The capacities of the police and criminal suspect to make an impact are also related to exogenous practices in the processing of interview data in the wider police investigation and legal process. Importantly, a local challenging of the power of the interviewer, or even recurring actions that undermine the role of the interviewer in these contexts, does not mean that the structural differences in the enduring capacities to act in such institutional interactions would necessarily dissolve.

The question authority of journalism, explored in the context of press conferences and the wider practices of news interviewing, provides a final example of how power in interaction is dependent on transformations of social roles and relations beyond the interaction. The research on press conferences (Clayman et al., 2010; Clayman and Heritage, 2021) has demonstrated how change in cultural and historical developments, and shifts in political regimes, can indeed inform the local design of social actions in interactions. Research has also shown that, in different cultures and political context, press conferences are organized in different ways, with decisive corollaries to interaction and discursive roles (Ekström and Eriksson, 2018). Journalists' opportunities to ask questions are regulated through pre-scheduled allocations of questions. In some institutional contexts, press conferences are strongly politically controlled allowing only a few pre-submitted questions. Moreover, extensive research has documented how the question authority of journalism in news interviews is challenged not only within the context of interviews but also through more general accusations of journalists and mainstream media in public discourse and populist propaganda, in the by-passing of mainstream journalism and the development of alternative contexts for mediated political discourse (Carlson et al., 2022). The multi-layered analysis of question authority thus requires CA research to be combined with other approaches to include exogenous social practices and institutional realities not manifest in interactions. The research has shown examples of how CA is combined with, for example, ethnography, but in the study of power, CA has not yet been applied integrated with other approaches. This is not to suggest an imposition of assumptions about structures on the analysis of the local interaction (Thornborrow, 2002; p. 18), but to account for exogenous social practices that create the structural conditions for journalists' power in and through the interaction.

## Discussion

We have now reviewed the contributions of CA to the sociological analysis of social power and—vice versa—the contributions of the sociological analysis of power to CA. We have argued for a concept of power in which the capacities of agents to influence and a make a difference by virtue of their social relations plays a central role. CA has already provided extensive evidence of how actors capacities to accomplish actions (*power-to*) and to govern and control others (*power-over*) are realized and negotiated in practices of talk and interaction. However, the precise role in which a participant's status of power in relation to their co-participant bears on action formation and ascription and the design of action in various activity contexts remains to be studied in the future.

Here, we predict that such effects will be found with reference to all key initiating actions indicative of a power relation (e.g., proposals, instructions, orders, commands, recommendations, requests). Thus, more specific hypotheses to be tested include, for example, that:

- In a speaker-tilted power relationship (i.e., the speaker has power over the recipient), the mere descriptions of past decisions and positive evaluations of currently available options are more likely to be treated as proposals by the recipients than in an equal or in a recipient-tilted power relationship.
- In a speaker-tilted power relationship, the presentation of ideas in the form of modal-conditional declaratives and interrogatives (i.e., archetypical ways of making a proposal) is more likely associated with commanding than in an equal or in a recipient-tilted power relationship.

Responsive actions, then again, may be assumed to vary with respect to the type and amount of resistance and “ownership” displayed in relation to the constraints previously imposed. Here, specific hypotheses to be tested include, for example, that:

- In a recipient-tilted power relationship (i.e., the recipient has power over the first speaker), vary passive forms of recipient resistance (e.g., silence) are more likely to lead the first speaker to account for their prior actions, compared to how such resistance would be treated in an equal or in a speaker-tilted power relationship.
- In a speaker-tilted power relationship, the recipients are more likely to try to “own” the decisions (e.g., complying while presenting independent reasons for the compliance) that the first speakers have unjustifiably imposed on them than the recipients in an equal or in a recipient-tilted power relationship would do.

Inasmuch as precise claims about the ways in which power is part of action formation and ascription can be made, the clearer becomes its role in the understanding of the sequential organization of action in interaction—a key topic of CA.

In this paper, we have suggested that CA's empirical focus on participants' observable actions in interactions does not need to assume a concept of power that is confined merely to actual behaviors and the outcomes of situated negotiations. On the contrary, our examples have shown how CA can contribute to an understanding of the multi-layered nature of the social relations by virtue of which actors have capacities to direct, govern, and control social interactions. Most crucially, we have argued for the sociological analyses of power that recognize the different realities of structures and agency and the emergent temporal dimensions of the structure/agency interdependency. While this notion constitutes a departure from the traditional idea of CA as a corrective alternative to sociological theorizing, we believe that the actual research within CA illustrates the importance of finding ways to move beyond the local negotiations of power toward a more encompassing view on the topic.

As sociologists we consider it important that also CA can engage in social and societal critique, and as the notion of power is an important tool to do so, we finally point to the options that a CA researcher, in our view, has in this respect. While power is obviously not always bad and destructive to those subject to and governed by it, power also creates inequalities, dominance, and oppression, which reduces the capacities and wellbeing of specific individuals and groups in society. To identify how power works, and what needs to change to counteract the negative forms of power, the sociological analysis of power, including CA, must focus on both the structural and institutional conditions of interaction and on what is going on locally in it. The analysis of power as an *antecedent* of social action, which can be observed in participants' orientations to their own and each other's accountabilities in various fields of action, allows us to identify, explore, and account for the enduring social realities that create inequal conditions for people. Likewise, the analysis of power as a *descendent* of interaction can shed light on the malleable and socially constructed nature of social reality and encourage the imagination of alternative futures with less inequalities and negative forms of power. In both ways, CA can be part of making the world into a better place.

## Author contributions

ME and MS have contributed to the same extent in all parts of the work behind this article. Both authors contributed to the article and approved the submitted version.
